# Using cheminformatics to predict cross reactivity of “designer drugs” to their currently available immunoassays

**DOI:** 10.1186/1758-2946-6-22

**Published:** 2014-05-10

**Authors:** Matthew D Krasowski, Sean Ekins

**Affiliations:** 1 Department of Pathology, University of Iowa Hospitals and Clinics, 200 Hawkins Drive, Iowa City, IA 52242, USA; 2 Collaborations in Chemistry, 5616 Hilltop Needmore Road, Fuquay-Varina, NC 27526, USA

**Keywords:** Amphetamines, Cannabinoids, Molecular models, Similarity, Toxicology

## Abstract

**Background:**

A challenge for drug of abuse testing is presented by ‘designer drugs’, compounds typically discovered by modifications of existing clinical drug classes such as amphetamines and cannabinoids. Drug of abuse screening immunoassays directed at amphetamine or methamphetamine only detect a small subset of designer amphetamine-like drugs, and those immunoassays designed for tetrahydrocannabinol metabolites generally do not cross-react with synthetic cannabinoids lacking the classic cannabinoid chemical backbone. This suggests complexity in understanding how to detect and identify whether a patient has taken a molecule of one class or another, impacting clinical care.

**Methods:**

Cross-reactivity data from immunoassays specifically targeting designer amphetamine-like and synthetic cannabinoid drugs was collected from multiple published sources, and virtual chemical libraries for molecular similarity analysis were built. The virtual library for synthetic cannabinoid analysis contained a total of 169 structures, while the virtual library for amphetamine-type stimulants contained 288 compounds. Two-dimensional (2D) similarity for each test compound was compared to the target molecule of the immunoassay undergoing analysis.

**Results:**

2D similarity differentiated between cross-reactive and non-cross-reactive compounds for immunoassays targeting mephedrone/methcathinone, 3,4-methylenedioxypyrovalerone, benzylpiperazine, mephentermine, and synthetic cannabinoids.

**Conclusions:**

In this study, we applied 2D molecular similarity analysis to the designer amphetamine-type stimulants and synthetic cannabinoids. Similarity calculations can be used to more efficiently decide which drugs and metabolites should be tested in cross-reactivity studies, as well as to design experiments and potentially predict antigens that would lead to immunoassays with cross reactivity for a broader array of designer drugs.

## Background

Immunoassays (antibody-based assays) are widely employed for drug of abuse/toxicology screening on urine or other bodily fluids. Immunoassays may utilize polyclonal or monoclonal antibodies, with a trend towards monoclonal antibody-based designs [1,2]. Many hospital-based clinical laboratories perform immunoassay drug of abuse screening panels targeted towards commonly abused drugs or drug classes such as amphetamines, benzodiazepines, cannabinoids, cocaine, methadone, opiates, and phencyclidine [3,4]. In addition to immunoassays, mass spectrometry-based methods such as gas chromatography/mass spectrometry (GC/MS) or liquid chromatography-tandem mass spectrometry (LC/MS/MS) can provide specific and definitive identification of drugs and drug metabolites; such methods are often used for confirmation of positive immunoassay screening results or for detection of drugs known to be undetectable or not readily detected by immunoassays [4-6]. While an increasing number of clinical laboratories are using mass spectrometry-based assays for drug of abuse testing, relatively few hospital-based clinical laboratories have the capability to do this testing with a rapid turnaround time. Thus, many clinical laboratories refer confirmatory testing to off-site regional or commercial reference laboratories, such that the turnaround time is too slow to aid in real-time patient management [3,4]. Consequently, immunoassays continue to be used in many settings for drug of abuse testing.

**Figure 1:**
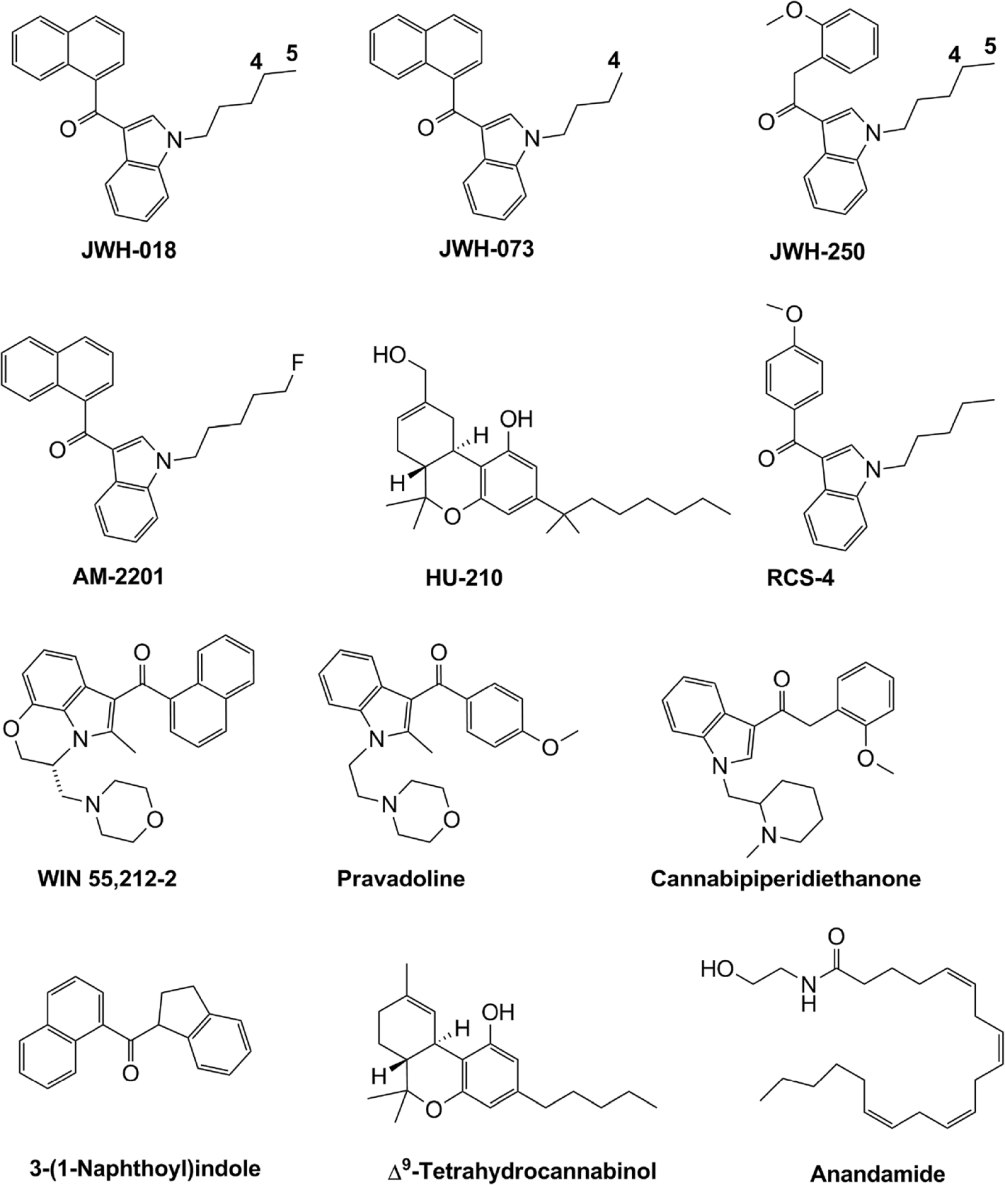
**Representative chemical structures of amphetamine-type drugs.** Abbreviations: MDMA, 3,4-methylenedioxy-*N*-methamphetamine; MDPV, 3,4-methylenedioxypyrovalerone; MDPBP, 3′,4′-methylenedioxy-α-pyrrolidinobutiophenone. Additional descriptions of compounds are in Additional file 1.

**Figure 2:**
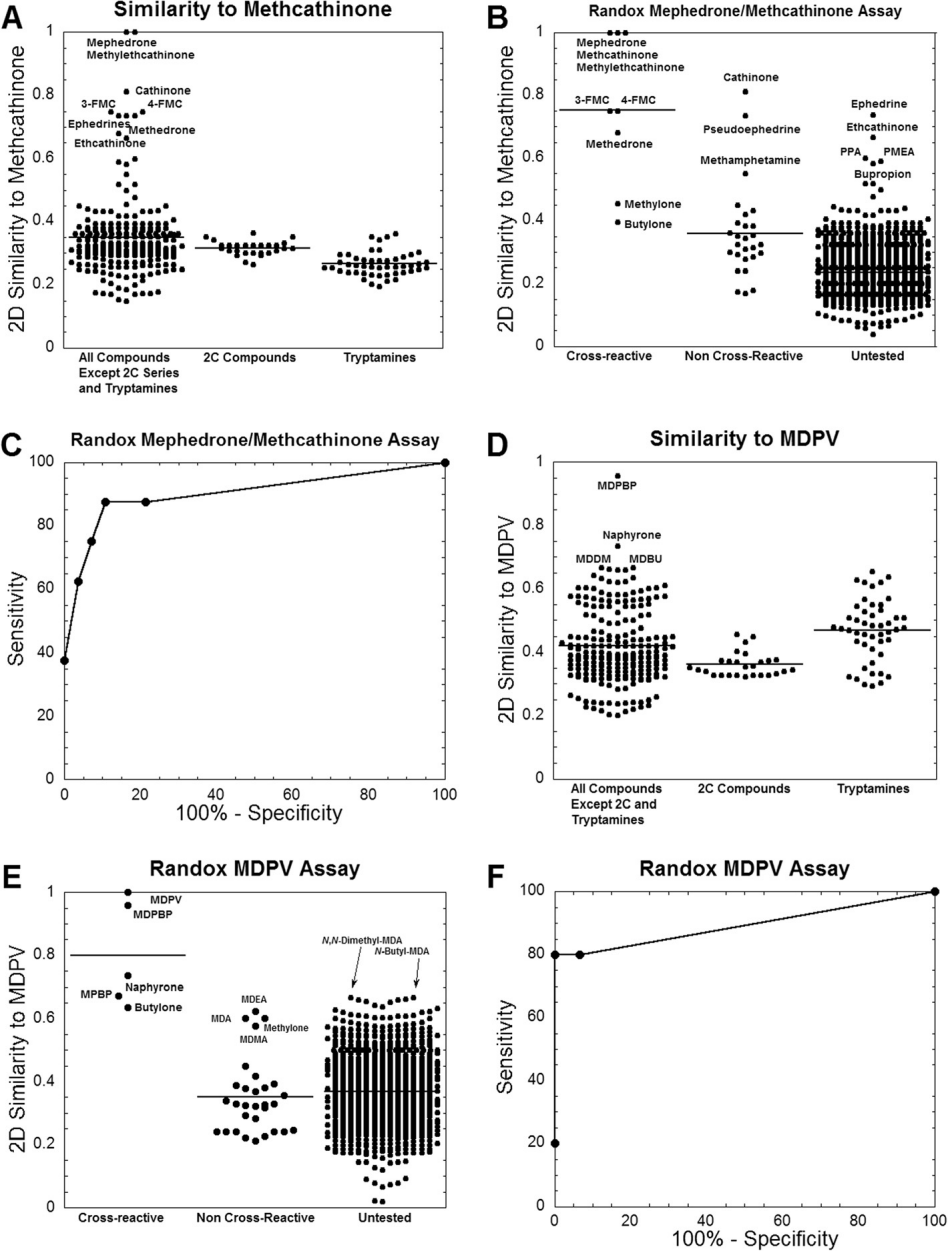
**Representative chemical structures of cannabinoids.** Detailed names for compounds are in Additional file 2.

A challenge for drug of abuse testing is presented by what are widely termed ‘designer drugs’, a heterogeneous group of psychoactive compounds typically discovered by modifications of existing clinical drug classes such as amphetamines [7-10]. Two current categories of designer drugs are the amphetamine-type stimulants and the synthetic cannabinoids, each of which comprises a chemically diverse set of compounds (see Figures 1 and 2 for representative compounds and their chemical structures). Designer drugs may be sold over the counter or via the internet using deceptive descriptors such as “plant fertilizer”, “incense”, “potpourri”, “research chemicals”, or “bath salts”. Further, many compounds are initially legal due to the regulatory challenge of trying to outlaw the large numbers of possible drug analogs that may be synthesized and distributed; authorities throughout the world have struggled with this issue [11,12]. In 2011, the United States Drug Enforcement Agency (DEA) temporarily classified mephedrone, 3,4-methylenedioxypyrovalerone (MDPV), and methylone as Schedule 1 drugs, a designation that indicates drugs with no accepted medical use and very high abuse liability [13]. Additionally, in June of 2012, the United States Congress approved Schedule 1 status for an additional 26 designer amphetamine-type stimulants and synthetic cannabinoids [14]. Clearly, due to the diversity of amphetamine-type and cannabinoid chemistry, there are likely many more structures that will ultimately require restricted status.

The amphetamine-type stimulants are related to amphetamine, methamphetamine, and 3,4-methylenedioxy-*N*-methylamphetamine (MDMA; “ecstasy”) [15]. Designer amphetamine-type drugs represent hundreds of individual compounds (many of which have detailed descriptions of chemical synthesis and psychoactive effects in two books by Shulgin and Shulgin [16,17]) and can be further broken down into sub-categories such as β-keto amphetamines, piperazines, tryptamines, and 2C compounds (see Figure 1 for examples). Methylone, MDPV, and mephedrone are the three most common amphetamine-like drugs currently referred to as “bath salts” and have received the most interest from the media and law enforcement [18,19].

The synthetic cannabinoids are similarly diverse and were originally examined for scientific and therapeutic applications (see Figure 2 for representative chemical structures) [20-22]. Like tetrahydrocannabinol (THC) from cannabis, the synthetic cannabinoids interact with cannabinoid receptors in the nervous system, producing a variety of psychoactive effects [23]. However, clandestine laboratories began to synthesize synthetic cannabinoids for use in products often marketed as “legal highs” [24]. Two of the most commonly abused synthetic cannabinoids are 1-naphthyl-(1-pentylindol-3-yl)methanone (JWH-018; named after discoverer John W. Huffman) and 2-(2-methoxyphenyl)-1-(1-pentylindol-3-yl) (JWH-250) [25]. As shown in Figure 2, some synthetic cannabinoids such as JWH-018 (a naphthoylindole) differ considerably in chemical structure from the “classic” cannabinoid chemical backbone of THC, while other compounds such as HU-210 (1,1-dimethylheptyl-11-hydroxy-THC) are analogs of THC.

Detection of designer amphetamine-type stimulants and synthetic cannabinoids in the clinical and forensic toxicology settings presents a complicated challenge [15]. There is a growing literature on detection of these designer molecules by mass spectrometry-based methods (see for example [26-40]). Drug of abuse screening immunoassays based on amphetamine, methamphetamine, and/or MDMA as the target molecule(s) cross-react with only a small subset of designer amphetamine-like drugs and are thus unreliable for detection of designer amphetamine-like drugs [41-45]. Immunoassays designed for THC metabolites (e.g., 11-nor-Δ^9^ THC-9-carboxylic acid, “9-carboxy-THC”) generally do not cross-react with the synthetic cannabinoids that do not share the classic cannabinoid backbone found in THC [15]. This suggests complexity in understanding how to detect and correctly identify whether a patient has taken a molecule of one class or another, and this ultimately impacts clinical care.

Recently, enzyme-linked immunosorbent assays (ELISAs) for “bath salts” [45] and synthetic cannabinoids [46] have been developed and analyzed for cross-reactivity. The use of immunoassays such as ELISA for detection of designer drugs raises the question of how well such assays will detect a variety of compounds while avoiding false positives caused by cross-reactivity with unrelated compounds. Two-dimensional (2D) molecular similarity analysis represents one of many potential cheminformatics approaches to this problem. In four previous publications, we provided proof of concept for the use of computational 2D or 3D similarity methods to predict cross-reactivity of compounds for immunoassays used for drug of abuse screening [44,47,48] and therapeutic drug monitoring [49] immunoassays. In these studies, 2D similarity using MDL keys/fingerprints were superior to 2D similarity using FCFP_6 fingerprints (one of many fingerprint types) and 3D pharmacophores in predicting cross-reactivity of immunoassays [44,47-49]. By comparing empirical data obtained from cross-reactivity studies with the molecular modeling studies, our published data indicate that 2D molecular similarity methods perform well in predicting cross-reactivity of drugs to existing drug of abuse screening immunoassays. Further, these methods can help prioritize compounds for cross-reactivity testing and identify novel cross-reacting compounds [48]. In this study, we now apply 2D molecular similarity analysis to the designer amphetamine-type stimulants and synthetic cannabinoids.

## Results

### Similarity analysis of amphetamine-like drugs

**Figure 3:**
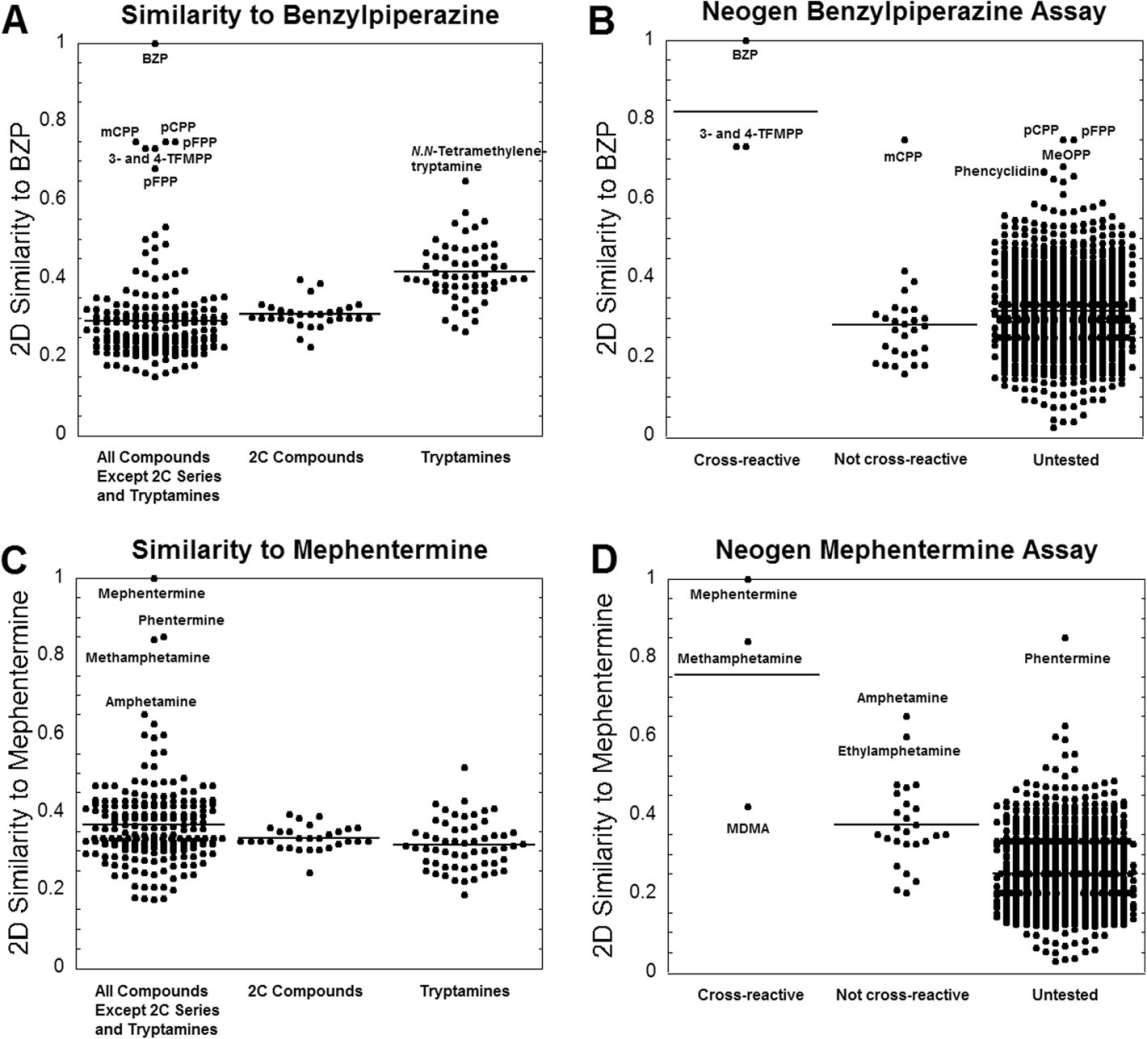
**Similarity analyses of the “bath salts” and prediction of immunoassay cross-reactivity.** Similarity analyses of mephedrone/methcathinone and 3,4-methylenedioxypyrovalerone (MDPV) – **(A)** 2D similarity of methcathinone to 287 amphetamine-like compounds. **(B)** 2D similarity of compounds that are cross-reactive, non-cross-reactive, or untested for the Randox Mephedrone/Methcathinone ELISA. **(C)** ROC curve analysis for the ability of 2D similarity to predict the cross-reactivity of compounds for the Randox Mephedrone/Methcathinone ELISA. The AUC is 0.942. Maximum efficiency of 88.9% is achieved at a cutoff of 0.455 (sensitivity = 87.5% and specificity = 89.3% at that cutoff). **(D)** 2D similarity of MDPV to 287 amphetamine-like compounds. **(E)** 2D similarity of compounds that are cross-reactive, non-cross-reactive, or untested for the Randox MDPV ELISA. **(F)** ROC curve analysis for the ability of 2D similarity to predict the cross-reactivity of compounds for the Randox MDPV ELISA. The AUC is 0.987. Maximum efficiency of 97.2% is achieved at a cutoff of 0.673 (sensitivity = 80.0% and specificity = 100.0% at that cutoff).

Mephedrone (4-methylmethcathinone) and MDPV are two common designer amphetamine-like drugs. Randox markets one ELISA assay targeting mephedrone/methcathinone and another assay targeting MDPV. Figure 3A shows the 2D similarity of methcathinone to 287 other amphetamine-like drugs (note: mephedrone and methcathinone have 2D similarity of 1.0 to each other due to how close they are in structure; see Additional file 1 for complete similarity and cross-reactivity data). The only molecules with 2D similarity of 0.6 or greater to methcathinone are other cathinone derivatives (e.g., methylethcathinone, 3-fluoromethcathinone). The 2C and tryptamine series of drugs all possess 2D similarity of less than 0.4 to methcathinone. Figure 3B shows the 2D similarity of compounds with cross-reactivity > 0.8% of methcathinone in the Randox Mephedrone/Methcathinone assay (cross-reactivity data is from both the assay package insert and publication by Swortwood et al. [45]) compared to non-cross-reactive compounds. None of the 2C and tryptamine compounds tested cross-react with the mephedrone/methcathinone ELISA. Six of the eight cross-reactive compounds have 2D similarity to methcathinone of greater than 0.68, whereas only two of twenty-five non-cross-reactive compounds have 2D similarity that high. Figure 3B also shows 2D similarity to methcathinone of the “untested” compounds (i.e., those compounds in Additional file 1 whose cross-reactivity has not been reported). Figure 3C shows ROC curve analysis of how well 2D similarity as a “diagnostic test” predicts cross-reactivity of compounds for the Mephedrone/Methcathinone assay. The area under the curve (AUC) is 0.942.

Figure 3D shows the 2D similarity of MDPV to 287 other amphetamine-like drugs (complete dataset is in Additional file 1). Only 23 other compounds in this dataset possess 2D similarity of 0.6 or greater to MDPV. Only four compounds (other than MDPV itself) cross-react with the Randox MDPV ELISA (cross-reactivity data is from both the assay package insert and publication by Swortwood et al. [45]). Figure 3E shows the 2D similarity of compounds with cross-reactivity > 0.8% of methcathinone in the Randox MDPV assay. All four cross-reacting compounds have 2D similarity greater than 0.6 to MDPV. Figure 3E also shows 2D similarity to MDPV of “untested” compounds. Figure 3F shows the ROC curve analysis of how well 2D similarity predicts cross-reactivity of compounds for the MDPV assay. The AUC is 0.987. Table 1 lists true positives, false positives, true negatives, and false negatives at select cutoffs for the assays depicted in Figure 3.

**Table 1:** True positives, false positives, true negatives, and false negatives for the immunoassays using selected 2D similarity cutoffs

**Assay**	**2D similarity cutoff**	**True positives**	**False positives**	**True negatives**	**False negatives**
Randox Mephedrone/Methcathinone	0.680	6	2	25	2
	0.390	8	6	19	0
Randox MDPV	0.630	5	0	30	0
Neogen Benzylpiperazine	0.730	3	1	28	0
Neogen Mephentermine	0.840	2	0	26	1
	0.420	3	7	19	0
JWH-018 Direct ELISA	0.673	27	21	44	3
JWH-250 Direct ELISA	0.875	3	0	92	0
Immunalysis MKT-1030	0.673	13	0	6	4
Immunalysis MKT-1032	0.673	15	0	3	4
Neogen Synthetic Cannabinoids	0.673	23	0	4	8

**Figure 4:**
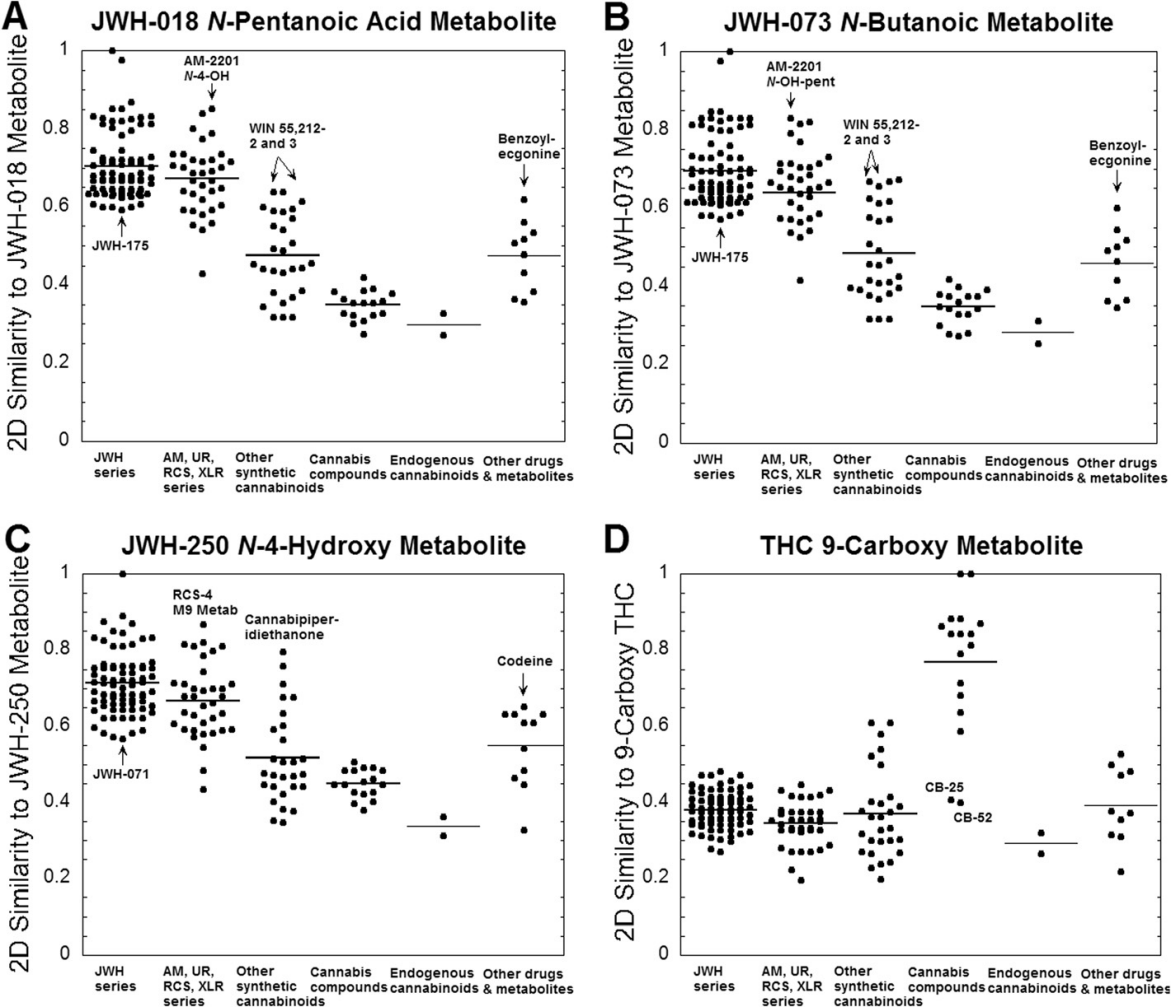
**Similarity analyses of benzylpiperazine (BZP) and mephentermine and prediction of immunoassay cross-reactivity. (A)** 2D similarity of BZP to 287 amphetamine-like compounds. **(B)** 2D similarity of compounds that are cross-reactive, non-cross-reactive, or untested for the Neogen Benzylpiperazine Forensic ELISA. **(C)** 2D similarity of mephentermine to 287 amphetamine-like compounds. **(D)** 2D similarity of compounds that are cross-reactive, non-cross-reactive, or untested for the Neogen Mephentermine Forensic ELISA.

Benzylpiperazine and mephentermine are two amphetamine-like drugs that may be abused [7,15]. Neogen markets separate ELISA assays for these two drugs. Figure 4A shows the 2D similarity of benzylpiperazine to 287 other amphetamine-like drugs (complete dataset is in Additional file 1). The only compounds with 2D similarity of 0.6 or greater are other piperazines, a single tryptamine compound (*N*,*N*-tetramethylenetryptamine), and phencyclidine. Of compounds tested for cross-reactivity, only two piperazine compounds (other than benzylpiperazine itself) cross-react with the Neogen Benzylpiperazine ELISA (Figure 4B; cross-reactivity data is from both the assay package insert and publication by Swortwood et al. [45]).

Figure 4C shows the 2D similarity of mephentermine to 287 other amphetamine-like drugs (complete dataset is in Additional file 1). The only compounds with 2D similarity to mephentermine of 0.6 or greater are phentermine, methamphetamine, amphetamine, *p*-methoxyethylamphetamine, 4-methylthioamphetamine, and ethylamphetamine. Only two compounds (methamphetamine and MDMA) cross-react with the Neogen Mephentermine ELISA (Figure 4D). Both of these compounds have 5% or less cross-reactivity relative to mephentermine [45]. MDMA cross-reacts but has low 2D similarity (0.421). Table 1 lists true positives, false positives, true negatives, and false negatives at select cutoffs for the assays depicted in Figure 4.

### Similarity analysis of synthetic cannabinoids

The synthetic cannabinoids are a diverse group of molecules with a nomenclature that can be confusing. Hundreds of compounds are in the JWH (John W. Huffman) series, although many have not yet been identified as drugs of abuse [22,24,25]. Additional file 2 has 43 JWH compounds of known toxicologic importance, along with 32 associated metabolites. Even within the JWH series are different classifications including naphthoylindoles (e.g., JWH-018), naphthylmethylindoles (e.g., JWH-175), and phenylacetylindoles (e.g., JWH-201). There are several other series of synthetic cannabinoids including the AM, UR, RCS, and XLR series; some of these are closely related to compounds in the JWH series [24,25].

**Figure 5:**
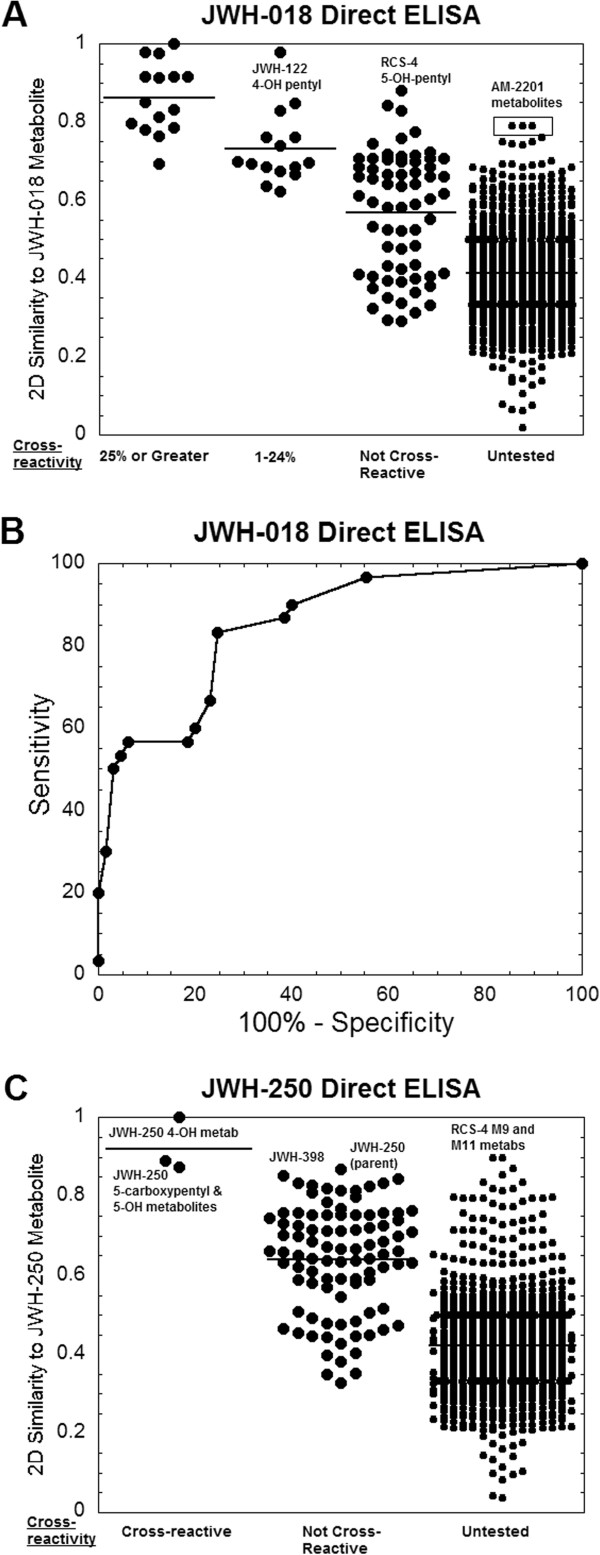
**Similarity comparisons of cannabinoids.** Test compounds are divided into broad categories (JWH series, AM/UR/RCS/XLR series, other synthetic cannabinoids not possessing the chemical backbone of THC, cannabinoids sharing chemical backbone of THC, endogenous eicosanoid cannabinoids, and non-cannabinoids). **(A)** 2D similarity of the *N*-pentanoic acid metabolite of JWH-018 to 168 other compounds. **(B)** 2D similarity of the *N*-butanoic acid metabolite of JWH-073 to 168 other compounds. **(C)** 2D similarity of the *N*-4-hydroxy metabolite of JWH-250 to 168 other compounds. **(D)** 2D similarity of 9-carboxy-THC to 168 other compounds.

Figure 5 shows plots of the 2D similarity of 168 other compounds compared to four cannabinoid targets, with test compounds divided into broad categories [(i)JWH series, (ii) AM/UR/RCS/XLR series, (iii) all other synthetic cannabinoids not possessing the classic cannabinoid backbone of THC, (iv) cannabinoids sharing classic cannabinoid backbone of THC, (v) endogenous eicosanoid cannabinoids, and (vi) non-cannabinoids]. Figure 5A-C uses metabolites of JWH-018, JWH-073, and JHW-250, respectively, as target molecules for the 2D similarity analysis. These metabolites were chosen because they are detected well by at least one commercial immunoassay for which cross-reactivity data is available (some compounds are the calibrator for the assay). Figure 5D uses 9-carboxy-THC as the target compound for 2D similarity analysis.

Figure 5A shows 2D similarity analysis using the *N*-pentanoic acid metabolite of JWH-018 (calibrator compound for the Immunalysis MKT-1030 and MKT-1032 assay kits) as the target. Within the JWH series (including metabolites), all but one other compound (JWH-175) have 2D similarity to the JWH-018 metabolite of 0.6 or greater. Outside the JWH series, a number of other compounds have high similarity. These include the *N*-4-hydroxy metabolite of AM-2201 (similarity = 0.851) and WIN 55,212-2/WIN 55,212-3 (both have similarity = 0.638). The compounds closely related to THC have low 2D similarity to the JWH-018 metabolite, as do the two endogenous cannabinoids (all with 2D similarity less than 0.420). A similar pattern is seen in Figure 5B with the JWH-073 *N*-butanoic acid metabolite (a metabolite detected well by the Immunalysis MKT-1030 and MKT-1032 kits). Figure 5C shows the 2D similarity to the *N*-*4*-hydroxy metabolite of JWH-250 (a calibrator for a JWH-250 ELISA that has been described in a publication by Arntson et al. [46]). The overall pattern of similarity is roughly that of JWH-018 and JWH-073 in Figure 5A and 5B, with the exception that pravadoline, metabolites of RCS-4, and cannabipiperidiethanone have high similarity to the JWH-250 metabolite (see Figure 2 for chemical structures). Figure 5D shows similarity of 9-carboxy-THC to 168 other compounds. Outside of compounds sharing the classic cannabinoid backbone of THC, the THC metabolite has generally low similarity to other cannabinoids. CB-25 and CB-52, which are in essence hybrids of THC and the endogenous cannabinoid anandamide, possess low 2D similarity (0.407 and 0.400, respectively) despite sharing some core features with THC.

**Figure 6:**
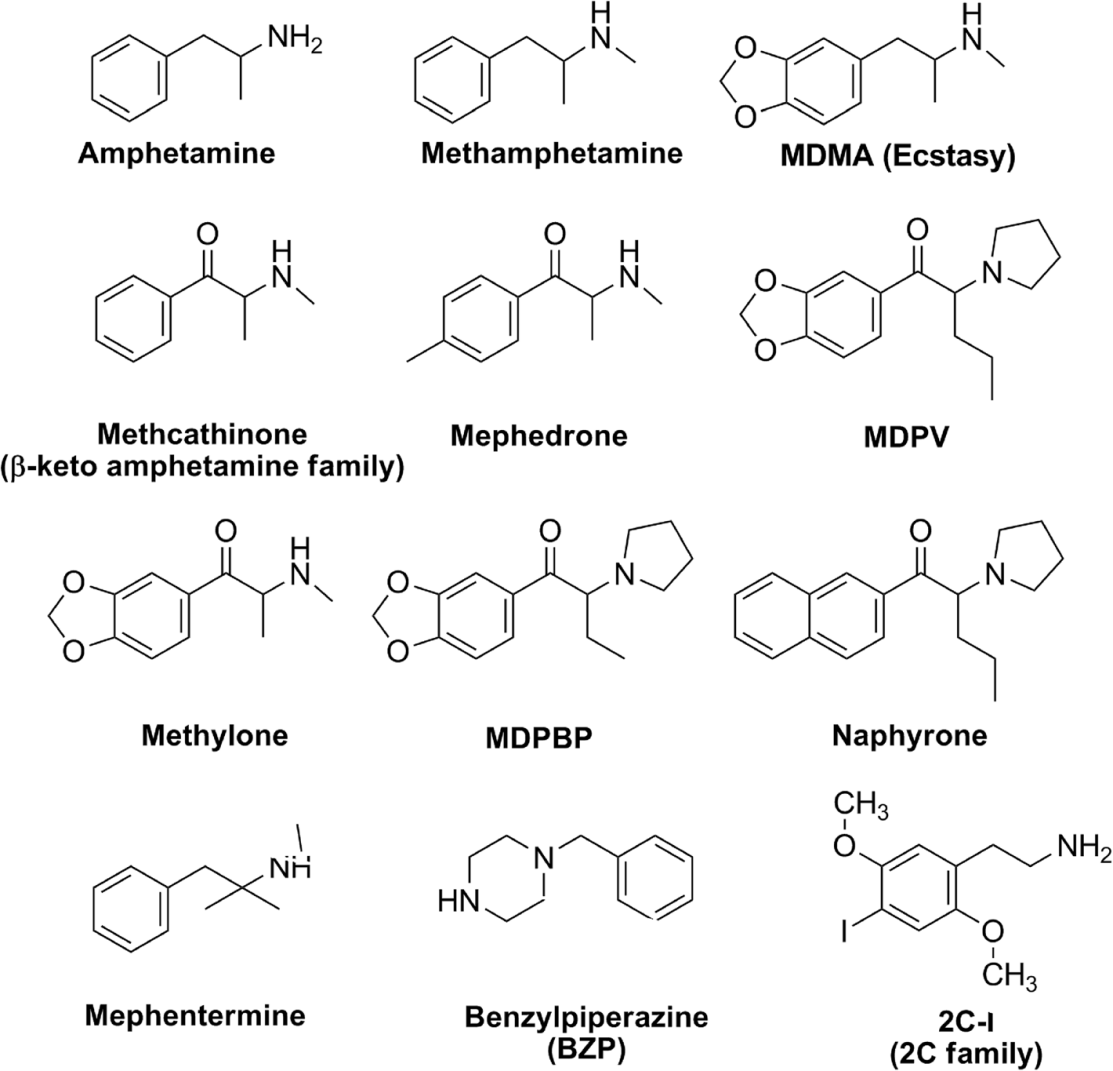
**Similarity analyses and prediction of immunoassay cross-reactivity for synthetic cannabinoids of the JWH series.** Similarity analyses for ELISA assays of JWH-018 and JWH-250 - **(A)** 2D similarity of compounds that are cross-reactive (divided into sub-categories of 25% or greater and 1-24%), non-cross-reactive, or untested for a JWH-018 Direct ELISA [46]. The 5-hydroxy metabolite of JWH-018 is used as the target for similarity analysis. **(B)** ROC curve analysis for the ability of 2D similarity to predict the cross-reactivity of compounds for the JWH-018 ELISA. The AUC is 0.987. Maximum efficiency of 82.1% is achieved at a cutoff of 0.673 (sensitivity = 93.8% and specificity = 56.7% at that cutoff). **(C)** 2D similarity of compounds that are cross-reactive, non-cross-reactive, or untested for a JWH-250 Direct ELISA [46]. The 4-hydroxy metabolite of JWH-250 is used as the target for similarity analysis.

Figure 6A shows cross-reactivity data of a JWH-018 ELISA which uses the 5-hydroxy metabolite of JWH-018 as the calibrator [46]. Using the published cross-reactivity data, the 2D similarity to the JWH-018 metabolite is displayed in Figure 6A for compounds with 25% or more cross-reactivity, 1-24% cross-reactivity, less than 1% cross-reactivity, or “untested” (i.e., using all remaining compounds from Additional file 2). All compounds with 1% or more cross-reactivity have similarity to the JWH-018 metabolite of 0.623 or greater. In contrast, similarity of 0.623 or higher was seen in only 30 of the 65 compounds that displayed cross-reactivity less than 1%. Figure 6B shows the ROC curve analysis of how well 2D similarity performed as a “diagnostic test” predicting cross-reactivity of the JWH-018 ELISA. The AUC is 0.859. Figure 6C shows cross-reactivity data of a JWH-250 ELISA which uses the 4-hydroxy metabolite of JWH-250 as the calibrator [46]. In this assay, only three metabolites other than the calibrator had cross-reactivity greater than 1%. The lowest 2D similarity for these three cross-reactive compounds was 0.875. Table 1 lists true positives, false positives, true negatives, and false negatives at select cutoffs for the assays depicted in Figure 6.

**Figure 7:**
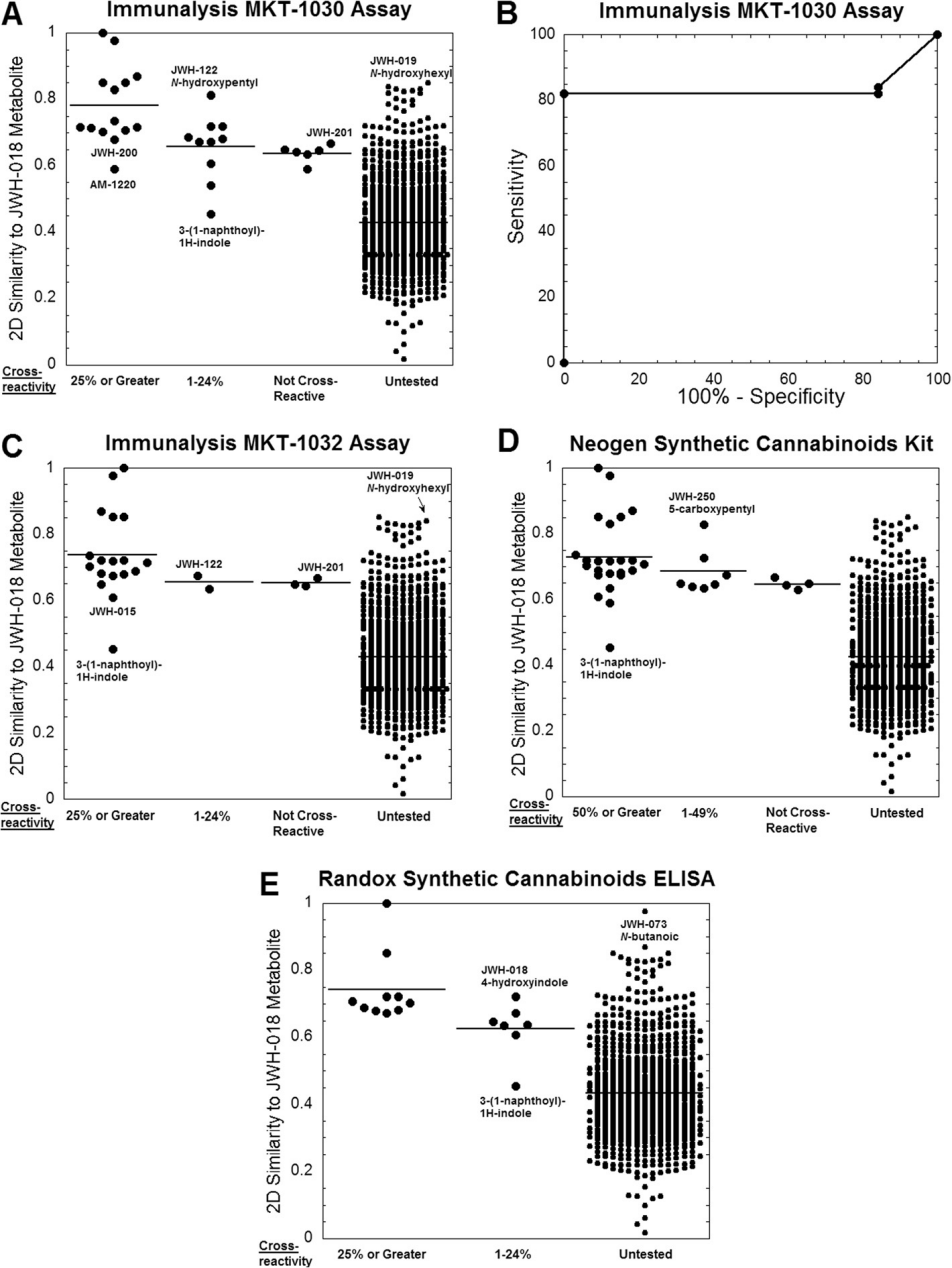
**Prediction of synthetic cannabinoid immunoassay cross-reactivity using 2D-similarity analysis.** Similarity analyses for synthetic cannabinoid immuonassays- **(A)** 2D similarity of compounds that are cross-reactive (divided into sub-categories of 25% or greater and 1-24%), non-cross-reactive, or untested for the Immunalysis MKT-1030 synthetic cannabinoid assay. The *N*-pentanoic acid metabolite of JWH-018 is used as the target for similarity analysis. **(B)** ROC curve analysis for the ability of 2D similarity to predict the cross-reactivity of compounds for the MKT-1030 assay. The AUC is 0.840. Maximum efficiency of 86.7% is achieved at a cutoff of 0.673 (sensitivity = 83.3% and specificity = 100% at that cutoff). **(C)** 2D similarity of compounds that are cross-reactive (divided into sub-categories of 25% or greater and 1-24%), non-cross-reactive, or untested for the Immunalysis MKT-1032 synthetic cannabinoid assay. The *N*-pentanoic acid metabolite of JWH-018 is used as the target for similarity analysis. **(D)** 2D similarity of compounds that are cross-reactive (divided into sub-categories of 50% or greater and 1-49%), non-cross-reactive, or untested for the Neogen Synthetic Cannabinoids assay. The *N*-pentanoic acid metabolite of JWH-018 is used as the target for similarity analysis. **(E)** 2D similarity of compounds that are cross-reactive (divided into sub-categories of 25% or greater and 1-24%) or untested for the Randox Synthetic Cannabinoids assay. The *N*-pentanoic acid metabolite of JWH-018 is used as the target for similarity analysis.

Figure 7A and 7C show cross-reactivity of the Immunalysis MKT-1030 and MKT-1032 synthetic cannabinoid assays, respectively, sorted by 2D similarity to the *N*-pentanoic acid metabolite of JWH-018 (calibrator for the assays). The highest 2D similarity of the non-cross-reactive compounds reported in the MKT-1030 and MKT-1032 package inserts to the JWH-018 metabolite was 0.667. For MKT-1030, only 1 of 14 compounds with 25% or more cross-reactivity and 2 of 12 compounds with 1-24% cross-reactivity had 2D similarity of 0.667 or lower (Figure 7A). For the MKT-1032 assay, only 4 of 18 compounds with 25% or more cross-reactivity had 2D similarity of 0.667 or lower (Figure 7C). Figure 7B shows the ROC curve analysis of how well 2D similarity performed as a diagnostic test predicting cross-reactivity of the MKT-1030 assay.

Figure 7D shows data for the Neogen Synthetic Cannabinoids assay. For this assay, the highest 2D similarity of the non-cross-reactive compounds to the *N*-pentanoic acid metabolite of JWH-018 was 0.667. Only 4 of 24 compounds with 50% or more cross-reactivity had a 2D similarity that low. 3-(1-naphthoyl)-1H-indole was unusual in having low 2D similarity to the JWH-018 metabolite (0.455) yet was cross-reactive in the Neogen and the two Immunalysis assays (Figure 7A,C,D). Figure 7E shows data for the Randox Synthetic Cannabinoids (Spice) ELISA kit. In this assay, all cross-reactive compounds had 2D similarity to the *N*-pentanoic acid metabolite of JWH-018 of 0.650 or higher. Only 2 of 7 non-cross-reactive compounds had similarity that high. Figure 7 also includes 2D similarity comparisons to untested compounds found in Additional file 2. Table 1 lists true positives, false positives, true negatives, and false negatives at select cutoffs for the assays depicted in Figure 7.

## Discussion

Previously we have shown in several studies that 2D molecular similarity methods perform well in predicting cross-reactivity of drugs to existing drug of abuse screening immunoassays [44,47,49]. In this study, we have compared published empirical data obtained from various cross-reactivity studies using MDL keys and 2D molecular similarity data in order to illustrate that such cheminformatics studies can predict cross-reactivity of designer amphetamine-type stimulants and synthetic cannabinoids. We have also been able to measure molecular similarity for many more compounds than have been tested experimentally for assay cross-reactivity including a large dataset of FDA approved drugs and their metabolites. In general, these compounds display 2D similarities below those of compounds cross-reactive with the immunoassays. We propose that molecular similarity can help differentiate between likely cross-reactive and non-cross reactive compounds to immunoassays for methcathinone/mephedrone, MDPV, and synthetic cannabinoids. This could be useful for selecting and classifying additional compounds that may also require DEA Schedule I status classification [13,14].

The evaluation and application of cheminformatics approaches to this research area has been limited [44,47-49]. We have previous compared FCFP_6 fingerprints (one of many fingerprint types) and 3D pharmacophores with MDL keys/fingerprints [44,48,49]. The goal of the current study was not to perform an exhausting analysis of fingerprints or similarity measures. Future studies could evaluate commercial and open source fingerprints (some of which may encode 3D information) as well as approaches to normalize, standardize, or combine data from different approaches using methods such as the belief theory [50] or data fusion [51-54]. In addition, approaches other than ROC curves could be used to evaluate the similarity and experimental cross-reactivity data [55].

A limitation of the similarity approaches is that these cannot account for the complex three-dimensional molecular interactions inherent in antibody-antigen binding. 3-(1-naphthoyl)-1H-indole is an example of a compound with low 2D similarity to the target compounds of an immunoassay, but which nonetheless has good cross-reactivity. 3-(1-naphthoyl)-1H-indole possesses the same overall shape as the JWH series of compounds but is missing a nitrogen atom along with the aliphatic tail (Figure 2). Compounds such as 3-(1-naphthoyl)-1H-indole may provide insight into the minimal sub-structural features important for antibody-drug interactions. An additional limitation of the similarity methods are that these do not account for concentration-dependence of cross-reactivity. There is very limited data on pharmacokinetics of synthetic cannabinoids and amphetamine-type drugs, especially at doses used to achieve intoxication. Even compounds with low cross-reactivity may be detected by an assay if present in urine or other body fluid at high concentrations.

As more experimental cross-reactivity data is generated for each immunoassay, it may be possible to use this to build machine learning models (e.g., Bayesian or Support Vector Machine) in order to predict this property for a new compound. The advantage of this approach is that it is not dependent on molecular similarity to a single molecule but instead uses the empirical data for a range of compounds for that subject of the immunoassay.

## Experimental

### Cross-reactivity data

Cross-reactivity data was found in multiple sources including published literature and package inserts for marketed assays. Randox Toxicology Limited (Crumlin, Antrim, United Kingdom) markets two ELISA kits for presumptive identification of “bath salts”, one targeting mephedrone/methcathinone (Mephedrone/Methcathinone ELISA, product # MD3475) and the other directed at detection of MDPV (MDPV ELISA, product # MD3476). Randox also markets an ELISA assay for synthetic cannabinoids [Synthetic Cannabinoids (Spice) ELISA, product # SC3474]. Immunalysis, Inc. (Pomona, CA, USA) markets two assays for synthetic cannabinoids [K2 (Synthetic Cannabinoids-1) Direct ELISA kit, product # MKT-1030; and Synthetic Cannabinoids (Spice, K2) Homogeneous Enzyme Immunoassay (HEIA), product # MKT-1032]. Neogen Corporation (Lexington, KY, USA) markets ELISAs for synthetic cannabinoids [Synthetic Cannabinoids (SPICE) ELISA kit], benzylpiperazine (Benzylpiperazine Forensic ELISA), and mephentermine (Mephentermine Forensic ELISA). Two publications report extensive cross-reactivity testing of immunoassays targeted at synthetic cannabinoids [46] and amphetamine-type stimulants [45].

For the Neogen Synthetic Cannabinoids ELISA, Randox Synthetic Cannabinoids ELISA, the Immunalysis synthetic cannabinoids assays, and two synthetic cannabinoids assays reported in the literature by Arntson et al. [46], there is extensive cross-reactivity data covering a wide range of values. Following an approach used in our previous publications [44,47-49], we have divided the data for these assays in different groups based on degree of cross-reactivity. The exact subdivisions are somewhat arbitrary (especially given the wide and varying numeric cross-reactivity values for these assays) but do provide a relative scale of strong versus weak cross-reactivity. All data is presented in dot plots that allow for visualization of the entire set of data.

### Virtual chemical libraries

The virtual chemical libraries (Additional files 1 and 2) used for molecular similarity analysis were built using parent drugs and metabolites identified in the package inserts and literature references cited in the previous paragraph and from additional published literature on pharmacokinetics of amphetamine-type stimulants [44,56-62] and synthetic cannabinoids [25,30,33,40,63-69]. The virtual library for synthetic cannabinoid analysis contained a total of 169 structures (including two endogenous eicosanoid cannabinoids – anandamide and 2-arachnidonylglycerol [70,71]); and (ten non-cannabinoids which have been tested for cross-reactivity in synthetic cannabinoid immunoassays [46]). The virtual library for amphetamine-type stimulants contained 288 compounds. Many of the amphetamine-type stimulants are described in two books by Shulgin and Shulgin [16,17] and the number used in these books is also included in Additional file 1 for cross-reference. Also included in the virtual chemical library was a database of Food and Drug Administration (FDA)-approved drugs (n = 676) derived from the Clinician’s Pocket Drug Reference [72], supplemented with drugs of abuse and drug metabolites (n = 110). This database has been used in four of our previous publications [48,49,73,74].

## Conclusions

In conclusion, we propose that similarity calculations can be used to more efficiently decide which drugs and metabolites should be tested in cross-reactivity studies, as well as to design experiments and potentially predict antigens that would lead to immunoassays with cross-reactivity for a broader array of designer drugs. As new analogs are synthesized and distributed, similarity calculations can guide and prioritize cross-reactivity studies. This very basic method may form the foundation for applying more complex cheminformatics approaches as more immunoassay cross-reactivity data is generated.

## Methods

### 2D molecular similarity analysis

Comparison of similarity of test molecules to the target compounds of the immunoassays in question used 2D similarity analysis, which determines the similarity between molecules independent of any *in vitro* data [75-77]. These methods have been applied in previous publications on cross-reactivity of drug of abuse and therapeutic drug monitoring immunoassays [44,47-49]. 2D similarity searching used the “find similar molecules by fingerprints” protocol in Discovery Studio versions 2.5.5 and 3.5 (Accelrys, Inc., San Diego, California, USA). MDL public keys (a specific 2D similarity algorithm) were used with an input query and with the Tanimoto similarity coefficient as the output (the coefficient ranges from 0 to 1, with 1 being maximally similar and 0 being maximally dissimilar; a comparison of a compound with itself or to a very closely related molecule can produce an output of 1). It should be noted that 2D similarity algorithms with this particular fingerprint method do not distinguish between diastereomers and enantiomers (although there are 2D similarity methods that can include stereoisomer information in generating fingerprint bits). There is very little experimental data on cross-reactivity of stereoisomers for the designer drugs and metabolites analyzed in this report. 2D similarity for each test compound was compared to the target molecule of the immunoassay undergoing analysis.

### Statistical analysis

Statistical analyses using receiver operating characteristic (ROC) curve analysis were carried out in EP Evaluator release 9 (Data Innovations, South Burlington, VA, USA). Sensitivity was defined as: (number of true positives)/(number of true positives + number of false negatives). Specificity was defined as: (number of true negatives)/(number of true negatives + number of false positives). Efficiency was defined as: (number of true positives + number of true negatives)/(number of true positives + number of true negatives + number of false positives + number of false negatives). ROC curve analysis plots the true positive rate (sensitivity) on the y-axis versus the false positive rate (1-specificity). EP Evaluator calculates the true and false positive rate at a range of thresholds for the 2D similarity in discriminating experimental determined assay cross-reactivity (positive) compared to lack of cross-reactivity (negative). ROC curve analysis was only performed if there were five or more cross-reactive compounds for a given assay. EP Evaluator does not allow for ROC curve analysis if less than five datapoints are available in either the positive or negative groups. This is to avoid erroneous conclusions based on ROC curve analysis of samples with small study size [78].

## Competing interests

The authors declare that they have no competing interests.

## Authors’ contributions

MDK and SE conceived and designed the experiments and analyzed the data. MDK wrote the initial manuscript. Both authors read and approved the final manuscript.

## Supplementary Material

Additional file 1 Cross-Reactivity and Similarity Data for Amphetamine-like Compounds: Contains common and scientific names and SMILES identifiers for compounds, along with cross-reactivity data of immunoassays and 2D similarity measures for the amphetamine-like compounds analyzed in this study. Click here for file

Additional file 2 Cross-Reactivity and Similarity Data for Cannabinoids: Contains common and scientific names and SMILES identifiers for compounds, along with cross-reactivity data of immunoassays and 2D similarity measures for the cannabinoid compounds analyzed in this study. Click here for file
